# Rampant Adaptive Evolution in Regions of Proteins with Unknown Function in *Drosophila simulans*

**DOI:** 10.1371/journal.pone.0001113

**Published:** 2007-10-31

**Authors:** Alisha K. Holloway, David J. Begun

**Affiliations:** 1 Section of Evolution and Ecology, University of California at Davis, Davis, California, United States of America; 2 Center for Population Biology, University of California at Davis, Davis, California, United States of America; Indiana University, United States of America

## Abstract

Adaptive protein evolution is pervasive in *Drosophila*. Genomic studies, thus far, have analyzed each protein as a single entity. However, the targets of adaptive events may be localized to particular parts of proteins, such as protein domains or regions involved in protein folding. We compared the population genetic mechanisms driving sequence polymorphism and divergence in defined protein domains and non-domain regions. Interestingly, we find that non-domain regions of proteins are more frequent targets of directional selection. Protein domains are also evolving under directional selection, but appear to be under stronger purifying selection than non-domain regions. Non-domain regions of proteins clearly play a major role in adaptive protein evolution on a genomic scale and merit future investigations of their functional properties.

## Introduction

Population genetics analyses indicate that protein divergence in Drosophila, unlike in humans and Arabidopsis, is frequently adaptive [1] (see review [2]). In flies, the proportion of amino acid substitutions that are adaptive has been estimated to be about 50% [1], [2] and is largely consistent across genes [3], [4]. Though most population genetics analyses of adaptive protein divergence treat entire proteins as single units, some analyses have addressed the question of the functional units within proteins that are the primary targets of directional selection (e.g. [5]–[7]). However, there are no genome-scale analyses addressing how population genetic processes may differ between functionally annotated regions of proteins versus those regions with no known function.

Protein domains serve a diversity of specialized functions relating to biochemical activity, binding affinity, subcellular location, or other aspects of protein biology. Regions of proteins that are not annotated as belonging to a domain may still have critical, yet unknown roles in protein function. This parsing of proteins raises the question as to which portion of proteins, domain vs. non-domain is more often subject to directional selection. In one world-view, if adaptive evolution implies functional divergence, such divergence might be more likely to occur in a known, functional domain. Alternatively, if most adaptive protein evolution resulted from fine scale tuning of function relating to, for example, protein folding, then adaptation might tend to occur in non-domain regions. Importantly, rates of divergence in annotated versus unannotated regions of proteins do not resolve these issues because variation in functional constraint cannot be distinguished from variation in the frequency of directional selection. We set out to investigate these issues on a whole-genome scale using population genetic data from the *Drosophila simulans* genome project.

## Results and Discussion

A syntenic assembly of partial genome sequences from six *D. simulans* lines was aligned to the reference sequence from the closely related species, *D. melanogaster* [1]. An alignment of the outgroup, *D. yakuba*, to *D. melanogaster* was used to infer substitutions specifically along the *D. simulans* lineage. Thus, the rich annotation of *D. melanogaster* was used directly to investigate polymorphism and divergence in *D. simulans*. *Drosophila melanogaster* annotations define the locations of many functional protein domains. We directly superimposed PROSITE domain coordinates (v4.3 *D. melanogaster* annotation; Table S1; [8]) onto the *D. simulans* population genomic data. Any codons that overlapped multiple domains were counted a single time. Overall, in these analyses we have data for 5,838 genes with defined domains that are comprised of 17,935 total domains, 1,013 of which are unique domain types.

**Figure 1:**
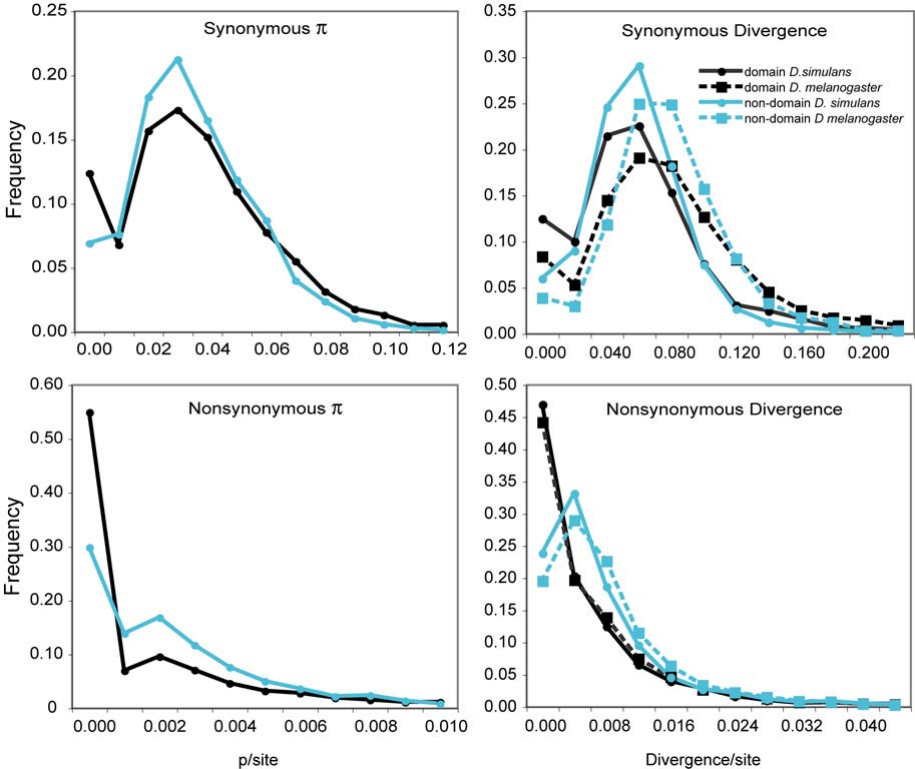
Distribution of polymorphism and divergence in domain and non-domain regions of proteins. Synonymous (top left panel) and nonsynonymous (bottom left) polymorphism in *D. simulans*. Lineage-specific divergence for synonymous (top right panel) and nonsynonymous (bottom right panel) sites in *D. simulans* and *D. melanogaster*.

We used contrasts of polymorphic and fixed, synonymous and nonsynonymous variants to compare the population genetics of domains to non-domain regions. Within genes, domain regions were concatenated and non-domain regions were concatenated for comparisons. These data can be found in Table S2 and data for polymorphism and divergence of each gene can be found in Table S3. Levels of synonymous polymorphism were similar between domains and non-domain regions (π_Sdom_ = 0.0338, π_Sout_ = 0.0333, for domains and non-domains, respectively; Mann-Whitney U [MWU] p = 0.0965; Figure 1). Rates of synonymous site divergence were also comparable (dS_dom_ = 0.0496, dS_out_ = 0.0502; MWU p = 0.0605; Figure 1). Amino acid polymorphism is quite similar, but is significantly lower in domains compared to non-domain regions (π_Ndom_ = 0.0020, π_Nout_ = 0.0022; MWU p<0.0001; Figure 1). The rate of protein evolution in domains was significantly lower than in non-domain regions (dN_dom_ = 0.0046, dN_out_ = 0.0055; MWU p<0.0001; Figure 1). Lower levels of protein polymorphism and divergence in domains are consistent with higher functional constraint. However, slower protein evolution could also result from less frequent adaptive evolution.

To distinguish between these alternatives we used the McDonald-Kreitman test [9], which tests the neutral theory prediction that the ratio of synonymous-to-nonsynonymous polymorphism should be the same as the ratio of synonymous-to-nonsynonymous divergence. Table 1 shows synonymous and nonsynonymous counts for codons in domains and non-domain regions (n = 4,969 genes). Domain and non-domain regions both reject the neutral model (Fisher's Exact Test [FET], p≪10^−6^). In both cases, the ratio of synonymous to nonsynonymous fixations is smaller than the corresponding ratio for polymorphism, which is consistent with adaptive protein divergence. Polymorphic and fixed synonymous variants in non-domain vs. domain sites are not significantly heterogeneous (1.82 vs. 1.81, FET p = 0.538; Table 1). However, the ratio of polymorphic-to-fixed nonsynonymous variants is significantly smaller for non-domain vs. domain codons (0.88 vs. 0.94, FET p = 0.008; Table 1). This suggests that although both classes of sites experience frequent adaptive fixation, non-domain codons may experience more adaptive evolution than domain codons.

**Table 1:** Sum of nonsynonymous and synonymous polymorphisms and fixations over domains and over non-domain regions.

Protein Region	Nonsynonymous			Synonymous			NI
	poly	fixation	poly:fix	poly	fixation	poly:fix	
Domain	4486	4773	0.94	30905	17095	1.81	0.52
Non-domain	11450	13002	0.88	64468	35406	1.82	0.48

FET: p-values = 0.008 and 0.538, for nonsynonymous and synonymous polymorphism (poly) to fixation (fix) ratios, respectively.

To investigate the distribution of variation on an individual gene basis, we used the neutrality index (NI), which is simply a different arrangement of McDonald-Kreitman 2×2 contingency tables [10]. Excess nonsynonymous fixation, one signature of adaptive protein evolution, causes NI to be less than 1. We retained 504 domain regions and 1,658 non-domain regions of genes that met our criteria of having at least five nonsynonymous and 5 synonymous variants for further analysis. One count was added to each cell in the 2x2 matrix in order to calculate NI in case any cell contained a zero. This procedure makes the test more conservative as adding one to each cell reduces the power to reject neutrality. Table S2 contains all counts of polymorphic and fixed variants used in analyses. We calculated NI for (1) codons within domains and (2) codons in non-domain regions (see Methods). The mean neutrality index in protein domains was significantly higher than non-domain regions (analysis of variance: p = 0.0030; both distributions were normally distributed after log_2_ transformation) indicating more frequent adaptive evolution in non-domain regions, which is consistent with the interpretation of the MK tests on pooled domain and non-domain codons. However, the proportion of codons in domains is much lower than in non-domain regions (35.7% vs. 64.3%; p<0.0001 MWU) and rates of amino acid divergence are slower. These two factors lead to many fewer counts being recorded in protein domains. Additionally, the method used to calculate NI (see Methods) is particularly conservative when counts are low. Given these limitations, we removed domain and non-domain regions with cell counts of zero for synonymous polymorphisms or fixations. We then recalculated NI without adding one to each cell. NI in non-domain regions is still lower than in domains, but not significantly so (analysis of variance: p = 0.0691).

In summary, both protein domains and amino acids in non-domain regions have experienced a high proportion of adaptive substitutions. Interestingly, non-domain regions appear to experience more frequent bouts of directional selection. This suggests that although non-domain regions may be less attractive targets of functional analysis in the laboratory, they are extremely important in terms of functional divergence under selection in nature. Future investigations of the mechanistic explanation of frequent adaptive evolution in non-domain regions, whether it is due to fine-tuning of folding patterns or yet to be discovered functions of non-domain regions, are clearly warranted.

## Materials and Methods

PROSITE protein domain coordinates from the *D. melanogaster* v4.3 annotation were retrieved by querying the ensembl database [8]. PROSITE domains were identified by the conservation of particular amino acid residues [11]. All domain coordinates for genes used in the analysis are listed in Table S1. Any codons that overlapped multiple domains were counted a single time.

Syntenic alignments of *D. simulans* and *D. yakuba* to the *D. melanogaster* reference are from [1]. Features were defined in the *D. melanogaster* v4.3 annotation from Flybase (ftp://ftp.flybase.net/genomes/Drosophila_melanogaster/dmel_r4.3_20060303/fasta). A single isoform from each gene (i.e. the isoform with the greatest number of codons) was used for analyses. We used a conservative set of genes that preserved the gene model of *D. melanogaster* in both *D. simulans* and *D. yakuba*. More specifically, the start codon and splice junction locations and sequence and the termination codon location agreed with the *D. melanogaster* reference sequence.

Polymorphism, as measured by nucleotide diversity (π), was estimated as in [1]. The numbers of silent and replacement sites were counted using the method of Nei and Gojobori [12]. The pathway between two codons was calculated as the average number of silent and replacement changes from all possible paths between the pair. Estimates of π on the X chromosome were corrected for sample size [π_ w_ = π * (4/3)] under the assumption that males and females have equal population sizes. Lineage-specific divergence was estimated by maximum likelihood using PAML v3.14 [13] and was reported as a weighted average over each *D. simulans* line with greater than 20 codons in the segment being analyzed. PAML was run in batch mode using a BioPerl wrapper [14] using codeml with codon frequencies estimated from the data. Table S3 contains all polymorphism and divergence estimates used in analyses.

For counts of polymorphic and fixed differences, we only analyzed codons where *D. melanogaster* and *D. yakuba* were identical. This allowed us to attribute fixed differences to the *D. simulans* lineage. Counts of nonsynonymous and synonymous polymorphisms and diverged sites took the path that minimized the number of nonsynonymous substitutions. All data were included in genomic comparisons of domains vs. non-domains. To be included in gene-by-gene domain vs. non-domain NI analyses, we required that there be at least 5 nonsynonymous variants and 5 synonymous variants for each domain/non-domain region. The neutrality index was calculated as the ratio of nonsynonymous polymorphisms to fixations divided by the ratio of synonymous polymorphisms to fixations [10]. One count was added to each cell in the 2x2 matrix in order to calculate NI in case any cell contained a zero. This procedure makes the test more conservative as adding one to each cell reduces the power to reject neutrality. Table S2 contains all counts of polymorphic and fixed variants used in analyses.

## Supporting Information

Table S1 PROSITE domain coordinates.
(0.47 MB TXT)

Table S2 Counts of polymorphic and fixed sites for (1) the portion of each gene in protein domains and (2) the remainder of the protein for each gene.
(0.39 MB TXT)

Table S3 Estimates of polymorphism and divergence for (1) the portion of each gene in protein domains and (2) the remainder of the protein for each gene.
(1.10 MB TXT)
